# Supplementary material for: Biophysical trade-offs in antibody evolution are resolved by conformation-mediated epistasis
Source: bioRxiv. 2026 Mar 16:2026.03.12.711465. Preprint. [Version 2] doi: 10.64898/2026.03.12.711465 (PMC13015495; doi:10.64898/2026.03.12.711465)
Supplement: Supplement 1 — Supplemental File 1. Plasmid map for integrating germline antibody into attb landing pad Supplemental File 2. Plasmid map for integrating Omi32 antibody into attb landing pad Supplemental File 3. Primers for combinatorial library generation Supplemental File 4. Primers for Illumina sequencing library preparation Supplemental File 5. Schematic of fluorescence-activated cell sorting for BioPhy-Seq measurements Supplemental File 6. Plasmid map for recombinant expression of germline antibody (light chain) Supplemental File 7. Plasmid map for recombinant expression of germline antibody (heavy chain) Supplemental File 8. Plasmid map for recombinant expression of Omi32 antibody (light chain) Supplemental File 9. Plasmid map for recombinant expression of Omi32 antibody (heavy chain) Supplemental File 10. Plasmid map for recombinant expression of BA1 spike trimer Supplemental File 11. Plasmid map for recombinant expression of BA4 spike trimer Supplemental File 12. Supplemental Tables 1–4 (cryo-electron microscopy imaging conditions and refinement statistics) Supplemental File 13. Video of antibody preconfiguration and antigen binding [file media-1.zip › SI files/Supplemental File 12.docx]

Supplemental File 12.

| **Supplemental Table 1. Grid and Glacios Imaging Conditions** | | | |
| --- | --- | --- | --- |
| **Sample** | **BA.1+germline+LCKappa** | **BA.4+germline+LCKappa** | **BA.4+Omi32+LCKappa** |
| Final Sample concentrations | 4 μM BA.1 (monomer), 8 μM germline, 16 μM LCKappa | 4 μM BA.4 (monomer), 20 μM germline, 40 μM LCKappa | 4 μM BA.1, 6 μM OmiGL, 12 μM LCKappa |
|  |  |  |  |
| Grid | UltrAuFoil 1/1 | UltrAuFoil 0.6/1 | UltrAuFoil 0.6/1 |
|  |  |  |  |
| Camera | Thermofisher Falcon 4 | Thermofisher Falcon 4 | Thermofisher Falcon 4 |
|  |  |  |  |
|  |  |  |  |
| Imaging Mode | Nanoprobe | Nanoprobe | Nanoprobe |
|  |  |  |  |
| Detector Mode | Counting | Counting | Counting |
|  |  |  |  |
| Electron Voltage | 200 kV | 200 kV | 200 kV |
|  |  |  |  |
| Nominal defocus | -1.0 μm to -2.5 μm | -1.0 μm to -2.5 μm | -1.0 μm to -2.5 μm |
|  |  |  |  |
| Nominal magnification | 150,000 | 150,000 | 150,000 |
|  |  |  |  |
| Pixel size | 0.923 | 0.936 | 0.936 |
|  |  |  |  |
| Total dose | 24.85 e^-^/Å^2^ | 25.05 e^-^/Å^2^ | 25.05 e^-^/Å^2^ |
|  |  |  |  |
| EER internal frames | 889 | 1491 | 1491 |
|  |  |  |  |
| Imaging | Image shift | Image shift | Image shift |
|  |  |  |  |
| Total # of images | 10,318 | 5,151 | 5,466 |

| **Supplemental Table 2. Grid and Krios Imaging Conditions** | | |
| --- | --- | --- |
| **Sample** | **germline+LCKappa** | **Omi32+LCKappa** |
| Final Sample concentration | 25 μM germline, 50 μM LCKappa | 25 μM Omi32, 50 μM LCKappa |
|  |  |  |
| Grid | UltrAuFoil 0.6/1 | UltrAuFoil 0.6/1 |
|  |  |  |
| Camera | Gatan K3 | Gatan K3 |
|  |  |  |
| Imaging Mode | Nanoprobe | Nanoprobe |
|  |  |  |
| Detector Mode | Counting | Counting |
|  |  |  |
| Electron Voltage | 300 kV | 300 kV |
|  |  |  |
| Nominal defocus | -0.5 μm to -2.0 μm | -0.5 μm to -2.0 μm |
|  |  |  |
| Nominal magnification | 165,000 | 165,000 |
|  |  |  |
| Pixel size | 0.512 | 0.512 |
|  |  |  |
| Total dose | 50.13 e^-^/Å^2^ | 50.54 e^-^/Å^2^ |
|  |  |  |
| Imaging | Image shift | Image shift |
|  |  |  |
| Total # of images | 8,731 | 8,653 |

| **Supplemental Table 3. Refinement Statistics** | | | | | | | | |  |
| --- | --- | --- | --- | --- | --- | --- | --- | --- | --- |
| **Sample** | **BA.1+germline+LCKappa** | | **BA.4+germline+LCKappa** | | | **BA.4+Omi32+LCKappa** | | |  |
| **PDB ID** | **11OQ** | | **11OO** | | | **11OL** | | |  |
| **EMDB ID** | **EMD-75891** | | **EMD-75889** | | | **EMD-75887** | | |  |
| Total extracted picks | 2,254,193 | | 1,811,974 | | | 2,320,037 | | |  |
| Final particles (no.) | 47,421 (C3 symmetry expanded) | | 160,551 | | | 106,182 | | |  |
| Symmetry | C1 | | C1 | | | C1 | | |  |
| FSC 0.143 | 3.2 Å | | 3.4 Å | | | 3.2 Å | | |  |
|  |  | |  | | |  | | |  |
| **Model** | | | | | | | | |  |
| Chains | 4 | | 4 | | | 4 | | |  |
| Atoms | 5673 (Hydrogens: 0) | | 5781 (Hydrogens: 0) | | | 5801 (Hydrogens: 0) | | |  |
| Residues | Protein: 735 Nucleotide: 0 | | Protein: 746 Nucleotide: 0 | | | Protein: 750 Nucleotide: 0 | | |  |
| Water | 0 | | 0 | | | 0 | | |  |
| Ligands | 0 | | NAG: 1 | | | NAG: 1 | | |  |
|  |  | |  | | |  | | |  |
| **Bonds (RMSD)** | | | | | | | | |  |
| Length (Å) (# > 4sigma) | 0.003 (0) | | 0.004 (0) | | | 0.005 (1) | | |  |
| Angles (°)(# > 4sigma) | 0.656 (1) | | 1.012 (0) | | | 1.024 (3) | | |  |
| MolProbity score | 1.74 | | 1.68 | | | 1.59 | | |  |
| Clash score | 8.36 | | 5.91 | | | 5.37 | | |  |
|  |  | |  | | |  | | |  |
| **Ramachandran plot (%)** | | | | | | | | |  |
| Outliers | 0.14 | | 0.14 | | | 0.13 | | |  |
| Allowed | 3.99 | | 5.03 | | | 4.18 | | |  |
| Favored | 95.87 | | 94.84 | | | 95.69 | | |  |
|  |  | |  | | |  | | |  |
| **Ramachandran plot Z-score (RMSD)** | | | | | | | | |  |
| Whole | -0.54 (0.32) | | -0.64 (0.32) | | | -0.31 (0.32) | | |  |
| Helix | -3.26 (0.58) | | -3.06 (0.61) | | | -1.81 (0.96) | | |  |
| Sheet | 0.06 (0.32) | | -0.32 (0.30) | | | -0.16 (0.33) | | |  |
| Loop | -0.17 (0.32) | | 0.02 (0.34) | | | 0.00 (0.31) | | |  |
| Rotamer outliers (%) | 0.00 | | 0.47 | | | 0.63 | | |  |
| Cbeta outliers (%) | NA | | NA | | | NA | | |  |
|  |  | |  | | |  | | |  |
| **Peptide plane (%)** | | | | | | | | |  |
| Cis proline/general | 13.2/0.0 | | 15.4/0.0 | | | 12.8/0.0 | | |  |
| Twisted proline/general | 0.0/0.0 | | 0.0/0.0 | | | 0.0/0.0 | | |  |
| CaBLAM outliers (%) | 2.36 | | 2.75 | | | 2.18 | | |  |
|  |  | |  | | |  | | |  |
| **ADP (B-factors)** | | | | | | | | |  |
| Iso/Aniso (#) | 5673/0 | | 5781/0 | | | 5801/0 | | |  |
| Min/max/mean |  | |  | | |  | | |  |
| Protein | 17.99/149.43/64.67 | | 19.69/178.47/75.13 | | | 12.70/172.38/73.73 | | |  |
| Nucleotide | --- | | --- | | | --- | | |  |
| Ligand | --- | | 112.59/142.42/134.31 | | | 68.63/104.95/95.22 | | |  |
| Water | --- | | --- | | | --- | | |  |
|  |  | |  | | |  | | |  |
| **Occupancy** | | | | | | | | |  |
| Mean | 1.00 | | 1.00 | | | 1.00 | | |  |
| occ = 1 (%) | 99.89 | | 99.57 | | | 99.52 | | |  |
| 0 < occ < 1 (%) | 0.11 | | 0.38 | | | 0.48 | | |  |
| occ > 1 (%) | 0.00 | | 0.00 | | | 0.00 | | |  |
|  |  | |  | | |  | | |  |
| **Data** |  | |  | | |  | | |  |
| Box |  | |  | | |  | | |  |
| Lengths (Å) | 81.97, 84.73, 115.12 | | 76.75, 77.69, 124.49 | | | 75.82, 83.30, 123.55 | | |  |
| Angles (°) | 90.00, 90.00, 90.00 | | 90.00, 90.00, 90.00 | | | 90.00, 90.00, 90.00 | | |  |
| Supplied resolution (Å) | 3.2 | | 3.3 | | | 3.2 | | |  |
| Resolution estimates (Å) | Masked | Unmasked | | Masked | Unmasked | | Masked | Unmasked | |
| d FSC (half maps; 0.143) | 3.2 | 3.4 | | 3.4 | 3.5 | | 3.3 | 3.4 | |
| d 99 (full/half1/half2) | 3.3/1.9/1.9 | 3.2/1.9/1.9 | | 3.6/1.9/1.9 | 3.5/1.9/1.9 | | 3.4/1.9/1.9 | 3.3/1.9/1.9 | |
| d model | 3.3 | 3.3 | | 3.6 | 3.6 | | 3.5 | 3.5 | |
| d FSC model (0/0.143/0.5) | 3.0/3.1/3.3 | 3.1/3.2/3.5 | | 3.2/3.3/3.6 | 3.3/3.4/3.7 | | 3.0/3.1/3.4 | 3.1/3.2/3.5 | |
| Map min/max/mean | -0.29/0.44/0.01 | | -0.40/0.72/0.00 | | | -0.35/0.54/0.00 | | |  |
|  |  | |  | | |  | | |  |
| **Model vs. Data** | | | | | | | | |  |
| CC (mask) | 0.81 | | 0.76 | | | 0.78 | | |  |
| CC (box) | 0.62 | | 0.62 | | | 0.59 | | |  |
| CC (peaks) | 0.56 | | 0.55 | | | 0.52 | | |  |
| CC (volume) | 0.79 | | 0.74 | | | 0.76 | | |  |
| Mean CC for ligands | --- | | 0.34 | | | 0.62 | | |  |

| **Supplemental Table 4. Refinement Statistics** | | | | | |  |
| --- | --- | --- | --- | --- | --- | --- |
| **Sample** | **germline+LCKappa** | | **Omi32+LCKappa** | | |  |
| **PDB ID** | **11OU** | | **11OR** | | |  |
| **EMDB ID** | **EMD-75893** | | **EMD-75892** | | |  |
| Total extracted picks | 2,740,105 | | 2,224,101 | | |  |
| Final particles (no.) | 73,477 | | 71,518 | | |  |
| Symmetry | C1 | | C1 | | |  |
| FSC 0.143 | 3.1 Å | | 3.2 Å | | |  |
|  |  | |  | | |  |
| **Model** |  | |  | | |  |
| Chains | 3 | | 3 | | |  |
| Atoms | 4166 (Hydrogens: 0) | | 4128 (Hydrogens: 0) | | |  |
| Residues | Protein: 549 Nucleotide: 0 | | Protein: 545 Nucleotide: 0 | | |  |
| Water | 0 | | 0 | | |  |
| Ligands | 0 | | 0 | | |  |
|  |  | |  | | |  |
| **Bonds (RMSD)** | | | | | |  |
| Length (Å) (# > 4sigma) | 0.002 (0) | | 0.003 (0) | | |  |
| Angles (°)(# > 4sigma) | 0.471 (0) | | 0.551 (0) | | |  |
| MolProbity score | 1.37 | | 1.50 | | |  |
| Clash score | 4.90 | | 7.54 | | |  |
|  | | | | | |  |
| **Ramachandran plot (%)** | | | | | |  |
| Outliers | 0.00 | | 0.00 | | |  |
| Allowed | 2.59 | | 2.42 | | |  |
| Favored | 97.41 | | 97.58 | | |  |
|  | | | | | |  |
| **Ramachandran plot Z-score (RMSD)** | | | | | |  |
| Whole | 0.48 (0.39) | | 0.31 (0.39) | | |  |
| Helix | 1.94 (1.37) | | 0.29 (1.13) | | |  |
| Sheet | 0.59 (0.35) | | 0.03 (0.34) | | |  |
| Loop | 0.09 (0.41) | | 0.60 (0.43) | | |  |
| Rotamer outliers (%) | 0.00 | | 0.89 | | |  |
| Cbeta outliers (%) | NA | | NA | | |  |
|  | | | | | |  |
| **Peptide plane (%)** | | | | | |  |
| Cis proline/general | 17.9/0.0 | | 17.9/0.0 | | |  |
| Twisted proline/general | 0.0/0.0 | | 0.0/0.0 | | |  |
| CaBLAM outliers (%) | 1.69 | | 1.51 | | |  |
|  | | | | | |  |
| **ADP (B-factors)** | | | | | |  |
| Iso/Aniso (#) | 4166/0 | | 4128/0 | | |  |
| Min/max/mean |  | |  | | |  |
| Protein | 11.91/116.02/55.48 | | 6.66/109.94/50.90 | | |  |
| Nucleotide | --- | | --- | | |  |
| Ligand | --- | | --- | | |  |
| Water | --- | | --- | | |  |
|  |  | |  | | |  |
| **Occupancy** | | | | | |  |
| Mean | 1.00 | | 1.00 | | |  |
| occ = 1 (%) | 99.93 | | 99.85 | | |  |
| 0 < occ < 1 (%) | 0.00 | | 0.15 | | |  |
| occ > 1 (%) | 0.00 | | 0.00 | | |  |
|  |  | |  | | |  |
| **Data** |  | |  | | |  |
| Box |  | |  | | |  |
| Lengths (Å) | 56.17, 76.41, 96.65 | | 56.17, 75.90, 94.62 | | |  |
| Angles (°) | 90.00, 90.00, 90.00 | | 90.00, 90.00, 90.00 | | |  |
| Supplied resolution (Å) | 3.2 | | 3.2 | | |  |
| Resolution estimates (Å) | Masked | Unmasked | | Masked | Unmasked | |
| d FSC (half maps; 0.143) | 3.2 | 3.3 | | 3.2 | 3.3 | |
| d 99 (full/half1/half2) | 3.3/1.0/1.0 | 3.2/1.0/1.0 | | 3.4/1.0/1.0 | 3.3/1.0/1.0 | |
| d model | 3.4 | 3.4 | | 3.4 | 3.4 | |
| d FSC model (0/0.143/0.5) | 3.0/3.1/3.3 | 3.0/3.1/3.4 | | 3.0/3.1/3.3 | 3.1/3.2/3.4 | |
| Map min/max/mean | -0.32/0.40/0.00 | | -0.25/0.41/0.01 | | |  |
|  |  | |  | | |  |
| **Model vs. Data** | | | | | |  |
| CC (mask) | 0.83 | | 0.83 | | |  |
| CC (box) | 0.66 | | 0.70 | | |  |
| CC (peaks) | 0.65 | | 0.68 | | |  |
| CC (volume) | 0.80 | | 0.80 | | |  |
| Mean CC for ligands | --- | | --- | | |  |
