# Supplementary material for: Biophysical trade-offs in antibody evolution are resolved by conformation-mediated epistasis
Source: bioRxiv. 2026 Mar 16:2026.03.12.711465. Preprint. [Version 2] doi: 10.64898/2026.03.12.711465 (PMC13015495; doi:10.64898/2026.03.12.711465)
Supplement: Supplement 1 — Supplemental File 1. Plasmid map for integrating germline antibody into attb landing pad Supplemental File 2. Plasmid map for integrating Omi32 antibody into attb landing pad Supplemental File 3. Primers for combinatorial library generation Supplemental File 4. Primers for Illumina sequencing library preparation Supplemental File 5. Schematic of fluorescence-activated cell sorting for BioPhy-Seq measurements Supplemental File 6. Plasmid map for recombinant expression of germline antibody (light chain) Supplemental File 7. Plasmid map for recombinant expression of germline antibody (heavy chain) Supplemental File 8. Plasmid map for recombinant expression of Omi32 antibody (light chain) Supplemental File 9. Plasmid map for recombinant expression of Omi32 antibody (heavy chain) Supplemental File 10. Plasmid map for recombinant expression of BA1 spike trimer Supplemental File 11. Plasmid map for recombinant expression of BA4 spike trimer Supplemental File 12. Supplemental Tables 1–4 (cryo-electron microscopy imaging conditions and refinement statistics) Supplemental File 13. Video of antibody preconfiguration and antigen binding [file media-1.zip › SI files/Supplemental File 5.pdf]

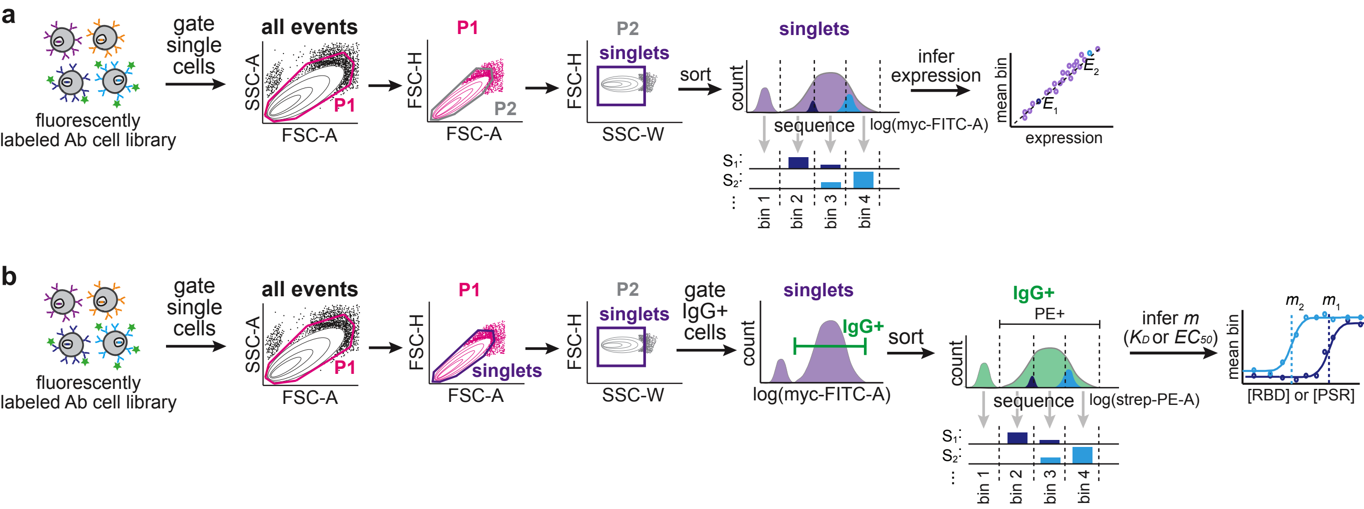

Sorting scheme for measurements of expression (a) and binding affinity and polyspecificity (b).

a, For expression, single cells were sorted into four, six, or eight populations based on FITC fluorescence intensity, with each gate capturing 25%, 16.7%, or 12.5% of the library, respectively.

b, For binding and polyspecificity, single IgG+ cells were sorted based on PE fluorescence using four gates – one containing non-binders and the other three containing 33% of the remaining population (low, medium, and high PE intensity).
