# Supplementary material for: Biophysical trade-offs in antibody evolution are resolved by conformation-mediated epistasis
Source: bioRxiv. 2026 Mar 16:2026.03.12.711465. Preprint. [Version 2] doi: 10.64898/2026.03.12.711465 (PMC13015495; doi:10.64898/2026.03.12.711465)
Supplement: Supplement 1 — Supplemental File 1. Plasmid map for integrating germline antibody into attb landing pad Supplemental File 2. Plasmid map for integrating Omi32 antibody into attb landing pad Supplemental File 3. Primers for combinatorial library generation Supplemental File 4. Primers for Illumina sequencing library preparation Supplemental File 5. Schematic of fluorescence-activated cell sorting for BioPhy-Seq measurements Supplemental File 6. Plasmid map for recombinant expression of germline antibody (light chain) Supplemental File 7. Plasmid map for recombinant expression of germline antibody (heavy chain) Supplemental File 8. Plasmid map for recombinant expression of Omi32 antibody (light chain) Supplemental File 9. Plasmid map for recombinant expression of Omi32 antibody (heavy chain) Supplemental File 10. Plasmid map for recombinant expression of BA1 spike trimer Supplemental File 11. Plasmid map for recombinant expression of BA4 spike trimer Supplemental File 12. Supplemental Tables 1–4 (cryo-electron microscopy imaging conditions and refinement statistics) Supplemental File 13. Video of antibody preconfiguration and antigen binding [file media-1.zip › SI files/Supplemental_File_13_Legend.docx]

**Supplemental Video 1. Antibody preconfiguration and antigen binding**. The HCDR2 and LCDR1 loops in the germline antibody (blue) undergo a conformational change upon acquiring the mutations present in Omi32 (red). Loops are shown in lighter shades of blue and red, with the HCDR2 on the left and the LCDR1 on the right. After preconfiguration, Omi32 binds RBD (grey; Omi32+BA4 is shown).
